# Regional Effects of Perceived Risks of Harm on Cigarette Smoking among U.S. High School Seniors: Evidence from Monitoring the Future

**DOI:** 10.3390/ijerph18179120

**Published:** 2021-08-29

**Authors:** Jorge Medina

**Affiliations:** Department of Economics, New Jersey City University, Jersey City, NJ 07305, USA; jmedina7@njcu.edu; Tel.: +1-201-200-2163

**Keywords:** perceived risk, cigarette, smoking, Monitoring the Future

## Abstract

Overall, there has been an increasing trend in the perceived risk of harm from smoking among U.S. high school seniors. However, these perceptions of risk have been falling in recent years. This study uses regional-level panel data from the Monitoring the Future survey and a fixed effects model to estimate the effect of perceived risk on three regional measurements of smoking behavior: consumption, lifetime prevalence, and daily smoking prevalence. Elasticity measurements at regional levels show that an increase in perceived risk decreases these regional measurements of smoking behavior. Moreover, the results show that, at regional levels, these measurements of smoking behavior are more responsive to changes in the perceived risk associated with smoking than to changes in the price of cigarettes.

## 1. Introduction

Cigarette smoking is one of the most preventable causes of cancer, heart disease, stroke, and lung diseases. According to the U.S. Department of Health and Human Services [1], there are more than 16 million people in the U.S. living with a disease caused by smoking. Despite its negative impact on health, the prevalence of cigarette smoking in the U.S. has remained significant. For instance, 249 billion cigarettes were sold in the U.S. in 2017, with companies such as Philip Morris USA, Reynolds American Inc., ITG Brands, and Ligget accounting for about 92% of cigarette sales [2]. Moreover, 34.2 million adults in the U.S. of at least 18 years of age (13.7%) smoked cigarettes in 2018 [3]. Among youths, nearly 5% of all high school students smoke cigarettes [4].

Attention to cigarette consumption among young people is important because a large number of smokers start smoking in adolescence [5,6,7,8]. Moreover, adolescents who begin smoking are likely to remain smokers as adults [9]. Furthermore, smoking during adolescence increases the risk of illegal drug use, low academic achievement, and other behavioral problems [10].

Economic theory assumes that individuals maximize utility subject to prices and income. Cigarette demand represents utility-maximization points of consumption for a smoker. A simplified demand for cigarettes is a function of the price of cigarettes, prices of other goods, income, and taste. Imperfect information prevents the consumption determined by the cigarette demand from being efficient. This means that a consumer that is not fully informed about the consumption of cigarettes cannot maximize utility. In the case of cigarette smoking, the lack of knowledge about health hazards associated with smoking will prevent the consumption of cigarettes from being optimal (efficient). This is particularly true among young smokers who might heavily discount future health issues, generate lower risk perceptions associated with smoking, and therefore consume more cigarettes than the perceived optimal quantity.

The literature on health behavior has focused on price and income elasticities of smoking [11,12]. Research on U.S. cigarette demand and its determinants suggests that increasing the price of cigarettes is less likely to reduce their consumption among heavy smokers due to nicotine addiction [13]. However, price is a more effective tool to decrease consumption among young adults. Younger people are more sensitive to changes in the price given that their expenditure on cigarettes represents a larger portion of their incomes [14,15]. Additionally, young smokers are more sensitive to changes in the price of cigarettes [12,16,17,18]. Researchers have also explored the role of perceived health risks from smoking. Berg et al. [19], Murphy-Hoefer et al. [20], and Weinstein [21] found that perceived health risks from smoking are associated with smoking status. Williams et al. [22] found that smokers with intentions to quit smoking had higher risk perceptions, suggesting that smokers with low motivations to quit could benefit from more information about the health risks associated with smoking. Arens et al. [23] found that more realistic risk perceptions of smoking play a role in protection against youth smoking. Moreover, Grevenstain et al. [24] found that increasing risk perceptions associated with smoking can modify smoking behavior.

Despite the literature already available, research on perceived risk is still relevant. In the U.S., there has been an overall increasing trend in the perceived risk of harm from smoking among high school seniors (12th graders). However, these perceptions of risk have been falling in recent years. Specifically, Johnston et al. [25] found that the proportion of high school seniors who consider smoking a pack of cigarettes per day a great risk increased in the late 1970s and the 1980s, fell in the 1990s, increased inconsistently in the following years, and has been falling again since 2015. Given this decline in risk perceptions in recent years, continued research on risk perceptions of harm from smoking is timely.

This study aims to make three main contributions to the risk perception literature. First, it uses public access data from the 1976–2018 Monitoring the Future (MTF) surveys. This dataset has attractive features including its longevity and consistency, which allow for the examination of early and recent risk perceptions from smoking. MTF also tracks different measurements of smoking behavior and it is highly regarded in the public health community. A limitation of the public-use MTF data is the lack of granular geographical indicators, which are needed to match individual-level MTF data with the income data from the Bureau of Economic Analysis (BEA) and the price data from The Tax Burden on Tobacco (TBT). To circumvent this limitation, I conducted this analysis at the regional level and incorporated the regional rate of perceived risk as a determinant of regional smoking behavior. Second, this study uses updated data to present new estimations of the impact of perceived risk of harm on smoking behavior among U.S. high school seniors and compute risk elasticities. This information is relevant for confirming that perceived risk continues to be a significant determinant of youth smoking behavior—especially in light of the recent decline in perceived risks from smoking. Moreover, risk elasticity estimates will help in the prediction of the effects of regional interventions aiming to increase regional rates of risk perceptions associated with smoking among this population. Third, this study also provides new regional estimates of the impact of price while controlling for perceived risks of harm associated with smoking. These estimates yield price elasticities that are useful in comparing the regional effect of perceive risk against the effect of price on smoking behavior among U.S. high school seniors.

## 2. Materials and Methods

### 2.1. Sample

Data on smoking behavior came from the 1976–2018 public-use MTF surveys. The data were collected through annual surveys of nationally representative samples that include public and private high school students in the U.S. Every year, data collection has taken place in 120–140 schools selected to provide a representative cross-section of students throughout the contiguous 48 U.S. states [26]. For this study, I focused on data from students in the 12th grade that are 18 years of age or older. (California, New Jersey, Oregon, and Maine passed a legislation to increase the minimum legal sale age for tobacco products to 21 prior to the federal minimum age of sale increase in 2019. All analyses were repeated excluding 2017–2018 data, which would exclude these four states given their effective legislation change dates. While not reported here, results (available upon request) showed very little change.)

A limitation of the public-use MTF datasets is that they do not provide geographical identifiers except for U.S. regions (Northeast, Midwest, South, and West). Given this limitation, and in order to merge these data with the income data from the BEA and the price data from TBT, I computed regional weighted averages, which yielded a panel dataset of 4 regions (N) across 43 years (T).

### 2.2. Measures

**Figure 1:**
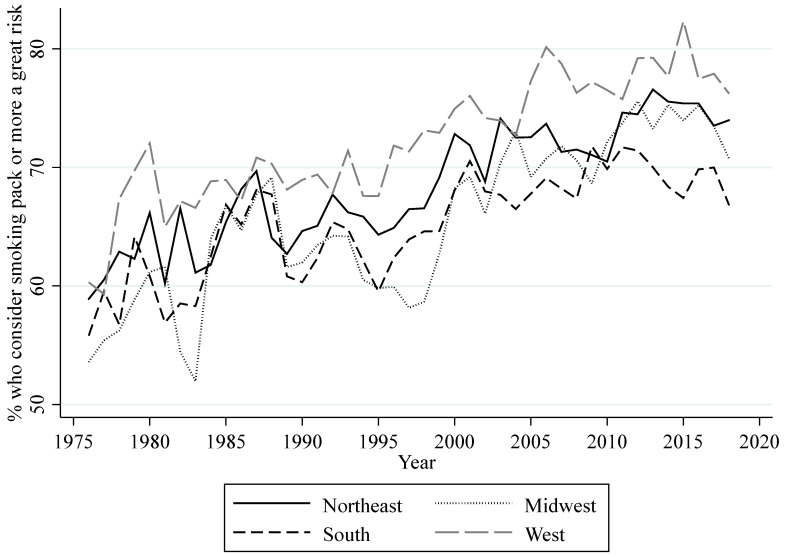
Annual percentage of U.S. high school seniors that consider smoking a great risk.

MTF provides data on the perceived risk of harm from smoking. In the MTF surveys, high school seniors answer how much they think people risk harming themselves if they smoke one or more packs of cigarettes per day. I used these data to compute regional annual percentages of high school seniors that consider smoking a great risk. (Computations are weighted averages for each region. Weights are provided by MTF.) Figure 1 shows the annual trends for this measurement for all regions. Overall, there is an increase in perceived risk of harm across regions, except for the decrease in the early to mid-1990s. Additionally, all four regions show a decline in perceived risk of harm among high school seniors since 2015.

**Figure 2:**
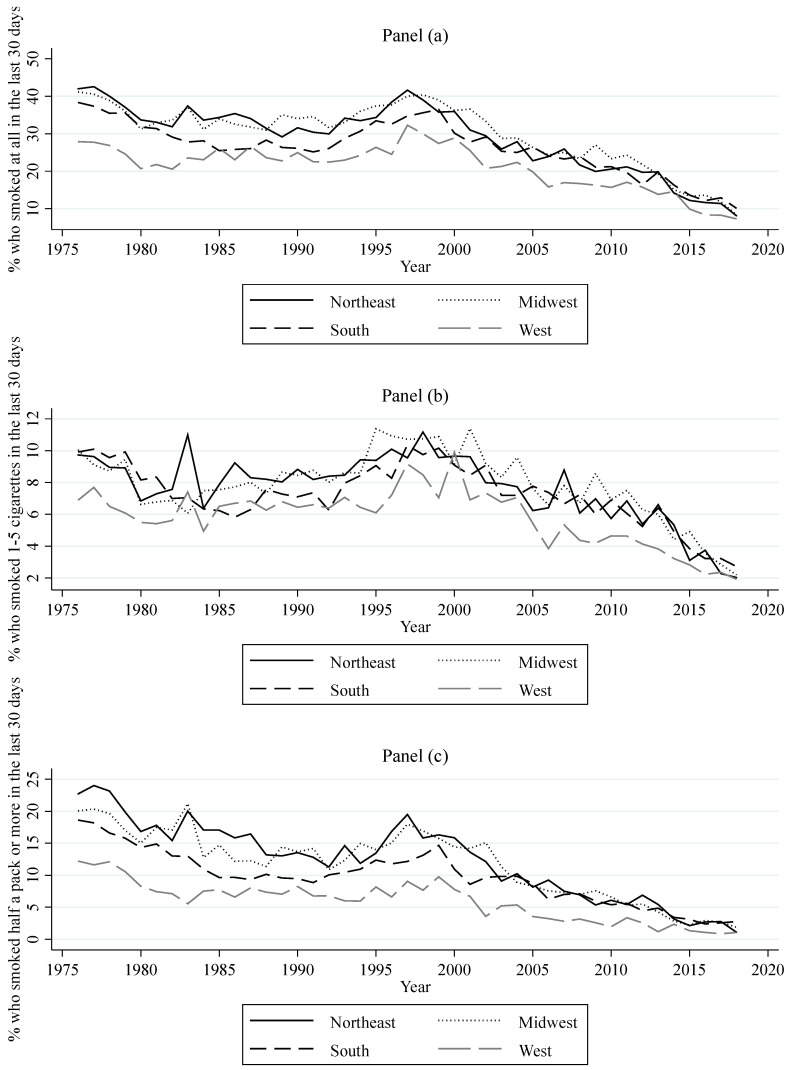
Annual prevalence rates for three categories (**a**–**c**) of consumption among U.S. high school seniors.

**Figure 3:**
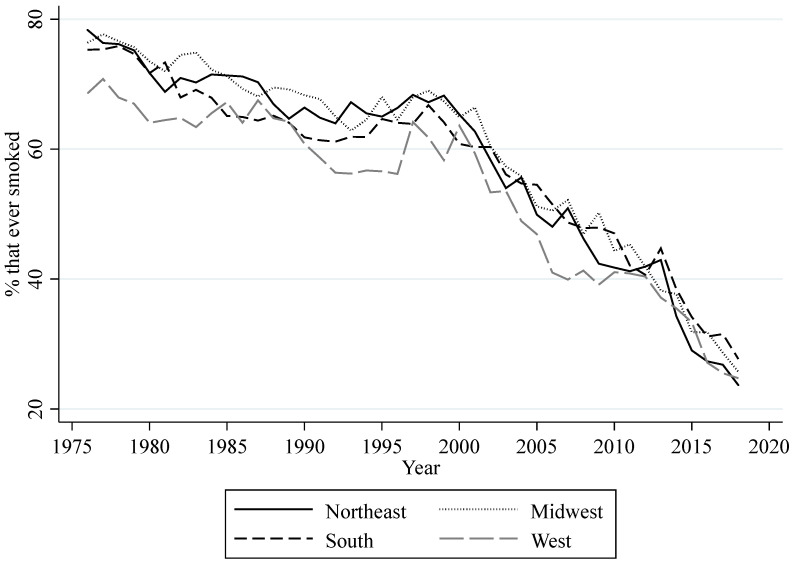
Annual rate of lifetime prevalence among U.S. high school seniors.

**Figure 4:**
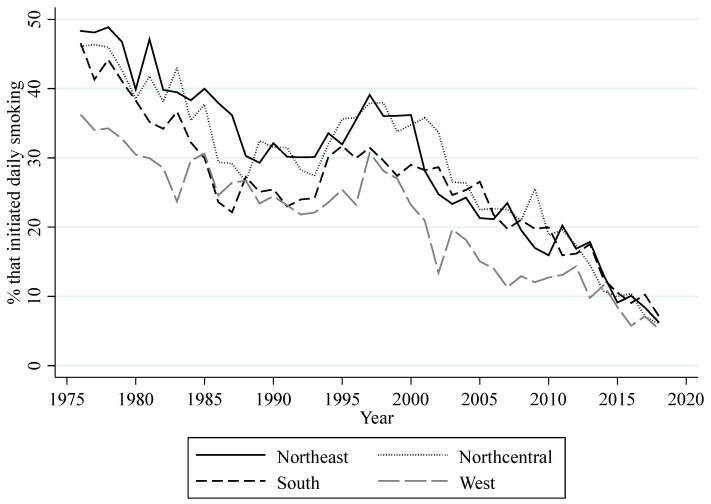
Annual percentage of U.S. high school seniors that have started daily smoking.

Data from respondents in the 12th grade MTF surveys who are at least 18 years of age were also used to compute different measurements of smoking behavior at regional levels. These measurements can be classified into measurements aiming to gauge (1) the prevalence of three different categories of regional consumption, (2) regional lifetime prevalence, and (3) regional daily smoking prevalence. The three categories of regional consumption are the regional percentages of high school seniors who, in the last 30 days, had a daily consumption of at least one cigarette (consumption), at most five cigarettes (light consumption), and half a pack or more (heavy consumption). The measurement of lifetime prevalence is the regional percentage of high school seniors that have ever smoked cigarettes (ever smoked). The measurement of daily smoking prevalence is the regional percentage of high school seniors that have started daily smoking (daily smoking). Figure 2 shows the yearly prevalence for the three categories of consumption for all regions. Overall, this figure shows a decrease in consumption, light consumption, and heavy consumption for each region. The decrease in these measurements follows through with the overall increase in perceived risk. When perceived risk decreased in the early to mid-1990s, all of these measurements of consumption increased. However, the recent decrease in perceived risk since 2015 seems to only have slowed down the decreasing trend in heavy consumption. Figure 3 shows the yearly percentage of lifetime prevalence for all regions. Similar to the measurements of regional consumption, the percentage of high school seniors that ever smoked a cigarette has declined throughout the years, with the exception of an increase around the mid-1990s. The decrease in perceived risk since 2015 appears to have slowed down the decreasing trend in this measurement, but only in the West. Figure 4 shows the yearly percentage of high school seniors that have started daily smoking. This figure also shows an overall decrease for daily smoking prevalence with an increase in the mid-1990s. The decreasing trend for daily smoking prevalence also appears to have slowed down following the decrease in perceived risk since 2015. MTF data on gender and race were used to compute annual regional percentages of high school seniors that are male and white for each year.

I also used BEA data on per capita disposable income and TBT data on the nominal yearly weighted average cost of a pack of cigarettes (not including taxes) to compute regional averages of these variables. (Population weights were calculated using BEA data.) Moreover, TBT publishes its data on cigarette prices in November, and MTF surveys take place between March and April of year t, with answers reflecting cigarette consumption in the past thirty days. For this reason, cigarette consumption is subject to the nominal cigarette price published in year t−1.

Table 1 shows U.S. regional averages across years for all the measurements of smoking behavior. Consumption and light consumption levels were the highest in the Midwest, whereas the Northeast had the highest percentage of high school seniors who were engaged in heavy consumption. In contrast, the West had the lowest average for all consumption categories. The average regional lifetime prevalence was also higher in the Midwest, but the average regional daily smoking prevalence was higher in the Northeast. Table 1 also shows average characteristics across years for each region. Perceived risk of harm associated with smoking among high school seniors was the highest in the West, while the South and the Midwest had the lowest average values of perceived risk. Price and per capita disposable income were the highest in the Northeast and the lowest in the South. Previous regional consumption among high school seniors was the highest in the Midwest and the lowest in the West. The percentage of male high school seniors was similar across regions but higher in the West, while the percentage of white high school seniors was the highest in the Midwest and the lowest in the South.

### 2.3. Methods

To estimate the effect of regional measures of risk and price on the regional smoking behavior among high school seniors across time, I used the following two-way linear fixed effects model:

(1) $$S_{it}={Χ}_{it}^{\prime }\beta +r_{i}+y_{t}+\varepsilon_{it}$$

where i=northeast, midwest, south, and west, and t=1976, 1977, …, 2018. In the model, $S_{it}$ stands for smoking behavior in region i and in year t, and it represents the five different measurements of regional smoking behavior explained in the previous section: consumption, light consumption, heavy consumption, ever smoked, and daily smoking.

The vector ${Χ}_{it}^{\prime }$ includes regional-level, time-varying characteristics such as the percentage of high school seniors who consider smoking a great risk ($R_{it}$), the average price of a pack of cigarettes ($P_{it}$), per capita disposable income ($I_{it}$), cigarette consumption in the previous year as a measurement for the habit related to smoking, the proportion of males, and the proportion of whites. $r_{i}$ and $y_{t}$ represent region and year fixed effects, and $\varepsilon_{it}$ is an error term. In the analysis, $P_{it}$ and $I_{it}$ are deflated by the 1982–1984 Consumer Price Index (CPI), which was taken from the Bureau of Labor Statistics.

The coefficients of $R_{it}$ were used to calculate risk elasticities, which measure the percentage change of all the measurements of smoking behavior resulting from a percentage change in perceived risk at regional mean values. Similarly, the coefficients of $P_{it}$ were used to calculate price elasticities, which measure the percentage change of all the measurements of smoking behavior resulting from a percentage change in the price of cigarettes (also at mean values).

The model assumes that the regressors in ${Χ}_{it}^{\prime }$ are exogenous but possibly correlated with the region- and time-invariant components of the error term. In the analysis, I obtained Driscoll–Kraay [27] standard errors to address issues of autocorrelation and correlation between regions. Driscoll–Kraay standard errors are robust to both temporal and cross-sectional correlations when T→∞, which is the case for the data in this analysis (N=4 and T=43). Moreover, measuring the habit related to smoking with a lagged value of consumption makes the data a dynamic panel when estimating the effects of the regressors on the daily consumption of at least one cigarette. However, according to Alvarez and Arellano [28], the fixed effects estimator bias introduced in this dynamic context is of the order 1/T. This argument makes estimators that correct for this bias unnecessary because it disappears given the relatively large time dimension of the data used in this analysis. This was confirmed by Arellano [29].

## 3. Results

Table 2 shows the estimations of equation 1. All dependent and independent variables are weighted averages measured at regional levels. This table also shows calculations of regional risk and price elasticities. The overall fitness of each specification is good with statistically significant F-values and R-squared values ranging from 0.879 to 0.992.

For the three categories of regional cigarette consumption, the main coefficients of interest (perceived risk and price) are significant, except in the specification for light consumption of cigarettes, where only habit has a statistically significant impact. Column (1) shows the results for consumption (daily consumption of at least one cigarette in the last 30 days). These results show that the risk coefficient yields a risk elasticity of −0.463, indicating that a 10% increase in the regional rate of perceived risk will decrease regional consumption by 4.63%. The price coefficient yields a price elasticity of −0.222. Additionally, income has a negative effect on consumption, showing that an increase in regional income is associated with a decrease in the regional percentage of high school seniors that consumed at least one cigarette per day. This is consistent with the evidence that people with a lower socioeconomic status tend to smoke more [30]. Habit agrees with previous findings [31] and reinforces the intuition that previous consumption increases current consumption. The coefficients for gender and race are not statistically significant. Column (2) shows the results for light consumption, which indicate that risk and price do not have a significant impact on this type of regional smoking behavior. Habit is the only significant determinant in this specification, suggesting that previous regional consumption also reinforces current light regional consumption of cigarettes.

Column (3) shows the results for heavy regional consumption of cigarettes, which indicate that, at regional levels, perceived risk and price are significant determinants of this type of smoking behavior. These estimations yield a risk elasticity of −1.333 and a price elasticity of −0.488. The income and habit coefficients confirm that both are also significant determinants of heavy consumption at regional levels. The coefficients for gender and race are not statistically significant.

For measurements of regional lifetime prevalence and daily smoking prevalence, perceived risk and price coefficients are significant. Column (4) shows that the coefficients for perceived risk and price yield elasticities suggesting that a 10% increase in each will decrease the regional percentage of high school seniors that have ever smoked by 2.34% and 1.39%, respectively. Results in column (5) show a risk elasticity equal to −0.506 and a price elasticity equal to −0.162. However, the coefficient for price is only significant at the 10% level, which could be due to the small cross-section dimension of the data. As pointed out by Gallet and List [12], the effect of price on cigarette consumption is sensitive to the data sample. Additionally, both specifications show that habit due to past consumption is significant and have a positive impact on the regional percentage that have ever tried smoking and have started daily smoking. The coefficient for gender is not statistically significant for regional lifetime prevalence or daily smoking prevalence. The coefficient for racesk is significant for daily smoking prevalence, but only at the 10% significance level.

## 4. Discussion

This paper used data from the 1976–2018 MTF public-use surveys to examine the effect of perceived risk on measurements of smoking behavior. The population of interest in this study was high school seniors (12th graders) in the U.S. The most prominent limitation of this study is the use of aggregated data at the regional level. The lack of granular geographical identifiers in the public-use MTF data is the reason for this limitation. Observations at the individual level only have geographical identifiers for U.S. regions (Northeast, Midwest, South, and West). This data limitation made it impossible to match individual-level data from MTF with the income data from the BEA and the price data from TBT. In order to circumvent this issue and still leverage the information from the public-use MTF surveys, regional weighted averages for all variables were computed, resulting in a regional panel dataset of 4 regions across 43 years. The use of regional data in this study yielded results that are only applicable at the regional level. Using these results to draw individual-level implications will lead to an incorrect assessment of the effect of perceived risk and price on smoking at individual levels.

Despite the limitations, the use of aggregated data can aid the examination of structural and contextual effects of human behavior [32]. In this study, the use of regional-level data provided a better understanding of the contextual effect of perceived risk on the different measurements of smoking behavior among high school seniors observed in this study. It also provided new estimations of the impact of price while controlling for risk, which allowed for a comparison with the impact of perceived risk.

The findings in this study show that regional rates of perceived risk and price have a statistically significant effect on two of the three categories of regional cigarette consumption among U.S. high school seniors: the regional percentages of high school seniors who, in the last 30 days, had a daily consumption of at least one cigarette (consumption) and half a pack or more (heavy consumption). Risk elasticity (−0.463) is larger (in absolute value) than price elasticity (−0.222) for consumption. Similarly, risk elasticity is also larger for heavy consumption (−1.333 vs. −0.488). These results indicate that the regional prevalence of high school seniors who had a daily consumption of at least one cigarette and of at least half a pack is more responsive to changes in perceived risk than to changes in price. In terms of practical significance, an increase in regional rates of perceived risk by 10% would reduce the regional consumption among high school seniors by 4.63% and regional heavy consumption by 13.33%. The same increase in price would decrease these measurements by 2.22% and heavy consumption by 4.88%, respectively. Furthermore, risk elasticity is the highest for regional heavy consumption, suggesting that this measurement may be particularly more responsive to changes in perceived risk.

For the regional percentage of high school seniors who smoked at most five cigarettes in the last 30 days (light consumption), regional rates of perceived risk and price do not have a statistically significant effect. Habit is the only statistically significant determinant. This is perhaps not surprising for the following reasons. Light smokers often do not identify themselves as smokers given their infrequent smoking [33]. This perception makes them susceptible to underestimating the risks associated with smoking [34], meaning that an increase in perceived risk would not have a significant impact on their smoking behavior. Moreover, young smokers who smoke at very low levels usually receive cigarettes from someone else [35]. Thus, an increase in price would not have a significant impact on the decision to smoke among light smokers.

Regional rates of perceived risk and price have a statistically significant impact on regional lifetime prevalence (ever smoked) among U.S. high school seniors. Increasing regional rates of perceived risk by 10% would reduce the regional percentage of high school seniors that have ever smoked by 2.34%, whereas the same increase in price would decrease this rate by 1.39%. Although this measurement of smoking behavior also appears to be more responsive to changes in regional rates of perceived risk, its impact is relatively small compared to the impact for consumption and heavy consumption.

In contrast, estimates of risk elasticity for the regional percentage of U.S. high school seniors that have started daily smoking are larger and equal to −0.506. In terms of practical significance, this result suggests that increasing regional risk perceptions among high school seniors by 10% would reduce the regional percentage that have started daily smoking by approximately 5%. The regional average price is also a statistically significant determinant of this regional measurement of smoking behavior, but not as strong in comparison to measurements of consumption and heavy consumption. This agrees with some findings in the literature. For instance, Douglas [36] found no correlation between cigarette prices and the decision to initiate smoking. Cawley et al. [37] found that cigarette prices are insignificant determinants of smoking initiation by young women. However, as pointed out by Gallet and List [12], this result could be due to data limitations such as the small cross-section dimension of the data used in this study. These data limitations can also explain why income is not a significant determinant of this regional measurement of smoking behavior.

These findings reveal the effect of risk perceptions of harm from smoking on cigarette consumption, lifetime prevalence, and daily smoking prevalence at regional levels. Regional risk perception describes a contextual environment that has an influence on these regional measurements of smoking behavior. These contextual effects have useful public health implications. Among U.S. high school seniors, the results in this study show that regions with higher rates of perceived risk have lower rates of cigarette consumption and heavy consumption, lower lifetime prevalence, and lower daily smoking prevalence (holding other variables constant). Effective interventions to reduce these measurements of smoking behavior would be through interventions at the regional level aiming to develop and disseminate accurate, credible, and age-appropriate information on the risks associated with cigarette smoking. Moreover, this study shows that risk elasticities are larger than price elasticities, suggesting that regional interventions should use rates of perceived risk as their principal tool to reduce these measurements of smoking behavior. Finally, regional risk elasticity estimates also suggest tailoring these efforts to target the regional prevalence of consumption and heavy consumption and regional daily smoking prevalence among this population.

## 5. Conclusions

The results in this study highlight the role of perceived risk on the smoking behavior among U.S. high school seniors at the regional level. Among this population, the results show that the regional rate of perceived risk is still a significant determinant of two categories of regional cigarette consumption, regional lifetime prevalence, and regional daily smoking prevalence. Risk elasticities are greater than price elasticities, suggesting that regional-level interventions should emphasize the use of regional rates of perceived risk over price to reduce these regional measurements of smoking behavior. However, further research is needed to explore the effect of perceived risk on smoking behavior at the individual level.

## Figures and Tables

**Table 1:** Summary statistics.

	All Regions			Northeast			Midwest			South			West		
	N	Mean	S.D.	N	Mean	S.D.	N	Mean	S.D.	N	Mean	S.D.	N	Mean	S.D.
*Dependent Variables*															
Consumption	172	26.66	8.385	43	29.02	8.986	43	30.04	8.476	43	26.39	7.038	43	21.20	6.010
Light consumption	172	7.151	2.133	43	7.656	2.138	43	7.837	2.145	43	7.278	1.906	43	5.834	1.785
Heavy consumption	172	9.940	5.423	43	12.38	6.055	43	11.80	5.438	43	9.679	4.170	43	5.905	3.122
Ever smoked	172	57.35	14.08	43	58.34	15.23	43	59.70	14.65	43	57.82	12.90	43	53.54	13.16
Daily smoking	172	33.00	23.92	43	35.61	23.86	43	35.32	23.54	43	32.78	23.66	43	28.28	24.73
*Independent Variables*															
Perceived risk	172	67.87	6.012	43	68.41	4.960	43	65.63	6.571	43	65.17	4.421	43	72.27	5.213
Price	168	1.675	0.738	42	1.87	0.928	42	1.67	0.737	42	1.514	0.583	42	1.637	0.636
Income	172	14,629	2636	43	15,553	2856	43	14,472	2611	43	13,756	2616	43	14,737	2189
Habit effect	168	27.09	7.989	42	29.52	8.473	42	30.55	7.864	42	26.78	6.642	42	21.53	5.67
Male	172	51.18	3.126	43	50.92	3.484	43	51.38	3.385	43	50.33	2.575	43	52.13	2.792
White	172	81.81	13.13	43	86.10	7.129	43	89.47	5.443	43	68.31	9.969	43	83.35	15.93

Note: Price and income are measured in dollars deflated by 1982–1984 CPI. All other variables are measured in percentages.

**Table 2:** Determinants of smoking behavior among U.S. high school seniors.

	(1)	(2)	(3)	(4)	(5)
	Consumption	Light Consumption	Heavy Consumption	Ever Smoked	Daily Smoking
Perceived risk	−0.182 **	−0.000	−0.195 **	−0.198 **	−0.246 ***
	[0.067]	[0.051]	[0.090]	[0.082]	[0.084]
Price	−3.528 **	−0.521	−2.895 **	−4.746 ***	−3.199 *
	[1.454]	[0.671]	[1.142]	[1.491]	[1.835]
Income	−0.001 **	0.000	−0.001 **	−0.000	−0.001
	[0.000]	[0.000]	[0.000]	[0.001]	[0.001]
Habit effect	0.339 ***	0.103 *	0.221 ***	0.247 *	0.503 ***
	[0.113]	[0.051]	[0.066]	[0.124]	[0.116]
Male	0.014	−0.006	0.026	−0.049	−0.000
	[0.071]	[0.035]	[0.065]	[0.062]	[0.106]
White	0.003	0.009	−0.032	0.038	0.090 *
	[0.031]	[0.018]	[0.024]	[0.039]	[0.052]
Constant	50.706 ***	−3.284	43.172 ***	57.702 ***	49.100 **
	[8.055]	[8.112]	[11.617]	[13.176]	[20.627]
Region and Year FE	Yes	Yes	Yes	Yes	Yes
F-value	251.6 ***	26.73 ***	30.99 ***	84.44 ***	25.63 ***
R-sq	0.965	0.879	0.950	0.986	0.992
Number of regions	4	4	4	4	4
Number of years	43	43	43	43	43
Observations	168	168	168	168	168
*Elasticities*					
Perceived risk	−0.463	−0.003	−1.333	−0.234	−0.506
Price	−0.222	−0.122	−0.488	−0.139	−0.162

Note: Discroll–Kraay standard errors reported in brackets. * Denotes statistical significance at the 10% level, ** at the 5% level, and *** at the 1% level. Risk and price elasticities are evaluated at mean values.

## Data Availability

Data on smoking behavior came from the 1976–2018 public-use MTF surveys.
